# Comparison of the Mechanical Properties Between the Convex and Concave Inner/Apical Surfaces of the Developing Cerebrum

**DOI:** 10.3389/fcell.2021.702068

**Published:** 2021-07-23

**Authors:** Arata Nagasaka, Takaki Miyata

**Affiliations:** ^1^Department of Anatomy and Cell Biology, Nagoya University Graduate School of Medicine, Nagoya, Japan; ^2^Division of Anatomy, Meikai University of School of Dentistry, Sakado, Japan

**Keywords:** neuroepithelium, apical surface, elasticity, tension, atomic force microscopy, actomyosin, cell density

## Abstract

The inner/apical surface of the embryonic brain wall is important as a major site for cell production by neural progenitor cells (NPCs). We compared the mechanical properties of the apical surfaces of two neighboring but morphologically distinct cerebral wall regions in mice from embryonic day (E) E12–E14. Through indentation measurement using atomic force microscopy (AFM), we first found that Young’s modulus was higher at a concave-shaped apical surface of the pallium than at a convex-shaped apical surface of the ganglionic eminence (GE). Further AFM analysis suggested that contribution of actomyosin as revealed with apical surface softening by blebbistatin and stiffness of dissociated NPCs were both comparable between pallium and GE, not accounting for the differential apical surface stiffness. We then found that the density of apices of NPCs was greater, with denser F-actin meshwork, in the apically stiffer pallium than in GE. A similar correlation was found between the decreasing density between E12 and E14 of NPC apices and the declining apical surface stiffness in the same period in both the pallium and the GE. Thus, one plausible explanation for the observed difference (pallium > GE) in apical surface stiffness may be differential densification of NPC apices. In laser ablation onto the apical surface, the convex-shaped GE apical surface showed quicker recoils of edges than the pallial apical surface did, with a milder inhibition of recoiling by blebbistatin than in pallium. This greater pre-stress in GE may provide an indication of how the initially apically concave wall then becomes an apically convex “eminence.”

## Introduction

During development, tissue morphogenesis requires the coordination of cell behaviors such as proliferation, differentiation, and migration. These behaviors are well known to respond to chemical signals (Briscoe and Small, 2015). However, there are an increasing number of studies showing that cells also respond to mechanical cues. For example, mechanical forces imposed by developing smooth muscle layers are necessary for the emergence of villi in the developing intestine (Shyer et al., 2013), and the interplay between tissue stress and cell intercalations contributes to limb bud morphogenesis (Lau et al., 2015). Similarly, the stiffness of the substrate affects the proliferation rates of vascular smooth muscle cells (Brown et al., 2005) and the differentiation of mesenchymal stem cells into several different cell types (Engler et al., 2006). Then, how do cells to form brain structures generate, sense, and utilize mechanical forces? Such an understanding would be supported by the assessment of the mechanical properties of developing brain walls. Measurement of tissue- or cell-level elasticity (stiffness) by indentation tests using atomic force microscopy (AFM) (Franze, 2011; Iwashita et al., 2014) and laser ablation to assess tissue tension (Campàs, 2016) are helpful for this purpose. The present study applies these techniques to the embryonic mouse cerebral wall, especially on its inner surface, which faces a fluid-containing space called the ventricle *in vivo*.

**FIGURE 1:**
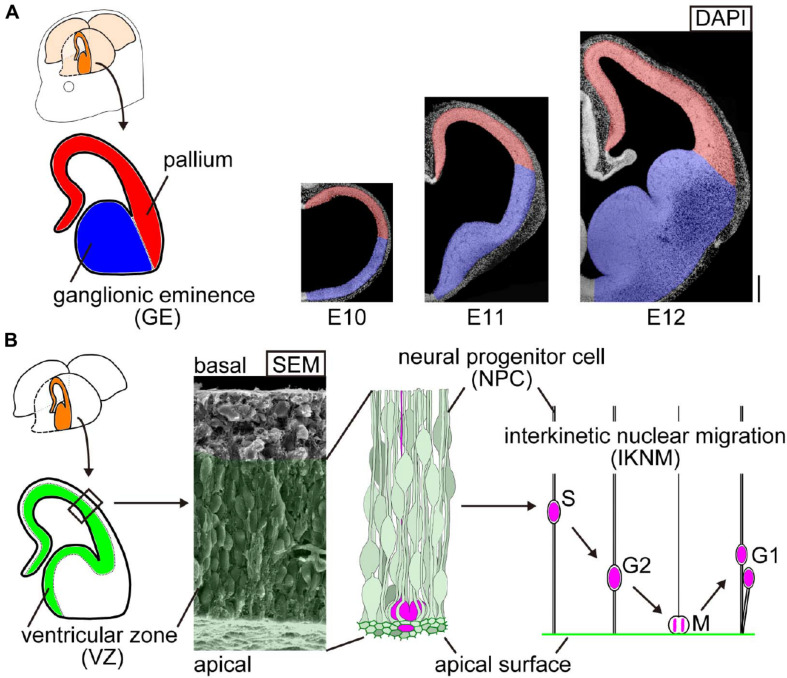
Schematic representation of the embryonic mouse cerebral wall. **(A)** Two morphologically distinct neighboring regions in the developing mouse cerebrum: the pallium (red, concave at the inner or apical surface) and the ganglionic eminence (GE) (blue, apically convex). Cerebral walls are all apically concave at embryonic day (E) 10, and convexity arises only in the GE domain by E12. Scale bar, 200 μm. **(B)** Composition and behavior of cells in the inner zone of cerebral walls. Elongated neural progenitor cells (NPCs) are tangentially assembled to form a wall. Their innermost (apical) portion is associated with each other via adherence junction. NPCs systematically move their nuclei and somata in a cell cycle-dependent manner: apical migration during G2 phase, followed by mitosis at the apical surface and further departure of daughter cells toward the basal side during G1 phase (called interkinetic nuclear migration, IKNM). The range of IKNM is histologically called the ventricular zone (VZ), which is filled with NPCs’ and their daughter cells’ nuclei/somata, as illustrated and shown in the scanning electron microscopy (SEM) image.

We were interested in a striking morphological difference between two neighboring cerebral domains in the mouse, which has become evident by embryonic day 12 (E12): a dorsal half called the pallium is concave at the inner (luminal, apical) surface, whereas a ventral half called the ganglionic eminence (GE) is apically convex (Figure 1A). Are the biomechanical properties of these inner surfaces different between these two regions at E12 and later, and if so, why? Can the emergence of these contrasting inner-surface morphologies (by E11) be explained by preexisting physical properties of the inner surface? Our preliminary trials revealed technical difficulties in currently addressing the latter question on events during the E10–E11 stage. Therefore, this study focused on the former questions and was designed to examine possible differences in mechanical properties between the concave and convex inner surfaces of E12–E14 cerebral walls and to further address the underlying cellular mechanisms.

The embryonic brain wall inner surface is important as a major site for cell production by neural progenitor cells (NPCs) (Figure 1B). NPCs are elongated and tangentially assembled to form a wall. Their innermost (apical) portion is associated with each other via adherens junction. Since the apex of each NPC endfoot is contractile in an actomyosin-dependent manner, the entire apical surface (mesh-like) is under tangential tension (Okamoto et al., 2013; Nagasaka et al., 2016). NPCs in a variety of brain regions and retina systematically move their nuclei and somata in a cell cycle-dependent manner: apical migration during G2 phase, followed by mitosis at the apical surface, and further departure of daughter cells toward the basal side during G1 phase (called interkinetic nuclear migration, IKNM) (Miyata, 2008; Taverna and Huttner, 2010; Strzyz et al., 2016). The range of IKNM is histologically called the ventricular zone (VZ).

We previously found links between NPCs’ IKNM behaviors and the physical properties of the VZ apical surface: (1) NPCs exhibited IKNM differently between mice and ferrets, and the apical surface of the ferret VZ was denser than that of the mouse VZ (Okamoto et al., 2014), while the elasticity (stiffness) of the AFM measured at the apical surface was greater in ferrets than in mice (Nagasaka et al., 2016); (2) the elasticity of the apical surface and the subapical space assist in the passive basal-ward departure of daughter cells (i.e., mechanical energy provided by an apically dividing NPC is transiently stored in the surrounding subapical space and elastically returned to its daughter cells) (Shinoda et al., 2018). These links and recently described differences in NPC behaviors between the pallium and the GE (Pilz et al., 2013) suggest that a comparative assessment of the mechanical properties of the apical surface of the pallium and that of the GE would establish a solid basis for better understanding of how morphologically different brain regions develop. Thus, we performed AFM-mediated indentation measurements at the apical surfaces of the E12–E14 pallial and GE walls in combination with pharmacological inhibition of actomyosin and laser ablation experiments.

## Materials and Methods

### Animals

Pregnant ICR mice were obtained from Japan SLC, Inc. Embryonic day zero (E0) was defined as the day of vaginal plug identification. For live visualization of the apical surface mesh comprised of VZ cell endfeet, we used a transgenic mouse line (R26-ZO1-EGFP: Accession no. CDB0260K) that ubiquitously expressed EGFP fused to mouse ZO-1 under the control of the *ROZA26* locus (Katsunuma et al., 2016). All protocols for animal experiments were approved by the Animal Care and Use Committee of Nagoya University.

### Preparation of Cerebral Walls for Atomic Force Microscopy Measurements at the Apical Surface of the Ventricular Zone

In measurement for the pallium, cerebral hemispheric walls (apicobasally 200–300 μm) were freshly isolated from embryos at E12–E14, processed (horizontally 400–500 μm × 400–500 μm, one to two pieces from a hemisphere) microsurgically in DMEM/F12, and transferred, with 1 ml of DMEM/F12, to a 35-mm dish previously covered partly with AteloCell IAC-30 collagen gel (Koken) at a concentration of 0.3 mg/ml. Pallial walls were gently placed with their apical surface facing up on top of the gel portion, which was approximately 5 mm thick and 20 mm in diameter (encircled by a silicone rubber ring attached to the dish surface). For the GE measurement, E12–E14 cerebral hemispheres were surgically dissected to remove the pallial region, and the remaining GE wall was gently placed (apical surface facing up) in a hole dug in solidified agarose gel (1%). The pallial and GE walls were completely submerged in DMEM/F12.

### Preparation of Dissociated Ventricular Zone Cells for Atomic Force Microscopy Measurement

Cerebral wall slices (coronal) from E13 embryos were microsurgically divided into an inner portion corresponding to the VZ and the remaining outer portion. The inner portion (VZ) was treated with trypsin-EDTA (0.05%) (Thermo Fisher Scientific). VZ cells were dissociated by gentle pipetting and plated on a polyethyleneimine-coated dish. Although the majority of the VZ cells were originally bipolar in shape and highly elongated (>100 μm long), the dissociation steps may have removed most of their long processes. AFM measurement was started 30 min after the cells were plated and finished within 60 min while cells were still rounded up with no spreading on the dish surface. We performed immunocytochemistry to these pallial or GE VZ cells. The percentage of Ki67-positive cells (i.e., progenitor cells) was 92 ± 3% in pallium and 92 ± 3% in GE (*n* = 3 VZs surgically removed from slices of E13 cerebral walls; the total number of cells counted were 290–697 cells per immunostaining following dissociation, plating, and immediate fixation of the VZ cells) (*p* = 0.83, Mann–Whitney *U*-test). The percentage of TUJ1-positive cells (i.e., neurons) was 11 ± 2% in pallium and 12 ± 3% in GE (*n* = 3 VZs surgically removed from slices of E13 cerebral walls; the total number of cells counted were 147–328 cells per immunostaining) (*p* = 0.83, Mann-Whitney *U*-test). This examination shows that our AFM measurements were performed on two VZ-cell groups (pallial and GE) that were almost equivalent in cell composition (mostly NPCs).

### Atomic Force Microscopy Indentation Measurements

All measurements were made with a Cellhesion200 (JPK Instruments) mounted on an IX71 inverted microscope (Olympus) equipped with a cantilever with a borosilicate bead (sQUBE, 20 μm in diameter for tissue and 5 μm in diameter for dissociated cells). The spring constant of each cantilever was determined before measurements were made using the thermal noise method (Hutter and Bechhoefer, 1993) in air (nominal value, 0.2 N/m). The applied forces were 10 nN for tissue and 1 nN for the dissociated cells. The approach and retraction velocities measured 5 μm/s for tissue and 1 μm/s for dissociated cells. Each measurement point was set in the central region of the apical surface of a cerebral wall or at the top of each dissociated cell. A force–distance curve was analyzed with the JPK DP software v.5 (JPK Instruments). Briefly, the Hertz model (Hertz, 1881) was applied to calculate Young’s modulus as follows:

$$F=\frac{E}{1-v^{2}}\,\left[\frac{a^{2}+R^{2}}{2}\,l\,n\,\frac{R+a}{R-a}-a\,R\right]$$

where *F* is the force, *E* is the Young’s modulus, *v* is the Poisson’s ratio, *a* is the radius of the contact circle, and *R* is the radius of sphere. The indentation depths were ∼4 μm for tissue and ∼1 μm for dissociated cells.

### Pharmacological Experiments

Cerebral walls and cerebral hemispheres from E13 embryos were treated with 1% DMSO (Sigma) (11 pieces from four embryos for pallium and 10 hemispheres from five embryos for GE), 20 μM blebbistatin (Calbiochem) (14 pieces from five embryos for pallium and 10 hemispheres from five embryos for GE), or 10 μM Y-27632 (Wako) (14 pieces from five embryos for pallium and 10 hemispheres from five embryos for GE) for 30 min before AFM measurement.

### Laser Ablation Experiments

Apices of neural progenitor cells were visualized by R26-ZO1-EGFP transgenic mice. Cerebral walls and cerebral hemispheres from E13 embryos were mounted with the apical side up in a plastic 35-mm dish (Corning). Laser ablation was performed with an IX81 inverted microscope (Olympus) equipped with CSU-X1 (Yokogawa), an iXon3 897 EMCCD camera (Andor), a 60× objective lens (UPLSAPO60XW, Olympus), an on-stage culture chamber (Tokai Hit) filled with 95% O_2_ and 5% CO_2_, and a MicroPoint (Andor) operated with iQ2 live cell imaging software (Andor). Theoretical spot size at the focal plane of the objective lens is 0.3 μm or less. With image acquisition in 0.5-s intervals, a pulse of 365-nm laser illumination at 16 Hz was simultaneously applied to the cell boundary at the apical surface.

### Imaging of Live Cerebral Walls and Cerebral Hemispheres

*En face* cultures of cerebral walls and cerebral hemispheres were prepared from R26-ZO1-EGFP transgenic mouse embryos. Cerebral walls and cerebral hemispheres from E12 to E14 embryos were mounted with the apical side up in a plastic 35-mm dish (Corning) using collagen gel. Confocal images were obtained on a BX51W1 microscope (Olympus) equipped with a CSU-W1 laser scanning confocal unit (Yokogawa) with a 100× objective lens (LUMPLFL100XW, Olympus) and an iXon+ EMCCD camera (Andor) in an on-stage culture chamber (TokaiHit) filled with 40% O_2_.

### Immunofluorescence

Cerebral hemispheres from E12 to E14 embryos were fixed in 4% paraformaldehyde. Frozen sections were treated with Alexa Fluor 488-conjugated phalloidine (Molecular Probes, 1:1,000) for 15 min at room temperature and subjected to confocal microscopy (FV1000; Olympus, Tokyo, Japan). Dissociated VZ cells were fixed with 4% paraformaldehyde in phosphate buffer (pH 7.4) and incubated with rabbit anti-Ki67 antibody (abcam, ab15580) or rabbiti-anti-neuron-specific beta III tubulin (TUJ1) (Covance, MRB-435P), followed by Alexa Fluor546-labeled anti-rabbit IgG antibody (Molecular Probes, 1:300).

### Measurement of the Spontaneous Narrowing/Shortening of the Apical Surface in Sliced Cerebral Walls

Cerebral walls from E12 mouse embryos were placed onto 35-mm dish (Corning) containing DMEM/F12 and recorded using an Olympus IX 71 (4×) equipped with an Orca ER camera (Hamamatsu Photonics) (*n* = 6 slices).

## Results

### The Apical Surface Was Stiffer in the Pallial Than in the Ganglionic Eminence

**FIGURE 2:**
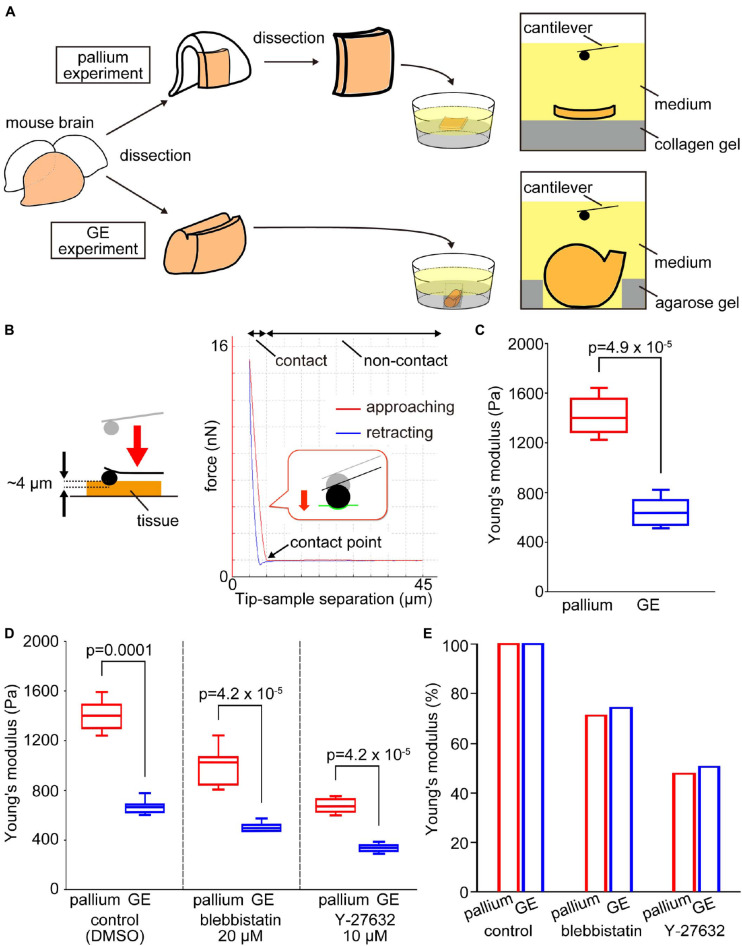
Experimental design and results of atomic force microscopy (AFM) measurements on the apical surface of the pallium and the GE. **(A)** Schematic diagram of the experiments. **(B)** Illustration showing an AFM indentation measurement on the apical surface of tissue (left) and a typical force–distance curve obtained (right). In the AFM indentation measurement of elasticity, the cantilever approach to the sample (red line), made contact with it, and then retract (blue line). The elasticity is calculated from the slope of the force–distance curve at the contact area of the red line. **(C)** Graph showing that the Young’s modulus measured vertically on the apical surface was greater for pallium (*n* = 11 pieces) than for GE (*n* = 12 hemispheres) (*p* = 4.9 × 10^–5^, Mann–Whitney *U*-test). **(D)** Graph showing the results of AFM indentation experiments in the presence of actomyosin inhibitors that affect the apical surface. Young’s modulus was reduced by inhibitors in both pallium and GE, but the pallium > GE relationship was maintained. **(E)** Graph showing the pallium–GE resemblance for the Young’s modulus reductions under actomyosin inhibitor conditions.

To compare mechanical properties at the apical surface between pallium and GE, we first performed indentation measurements to infer the stiffness using AFM. Fresh cerebral walls were prepared from embryonic mice at E13 and were placed on gel-coated dishes with the apical surface facing up (Figure 2A). The apical surface was pushed with a spherical bead (20 μm diameter) attached to the tip of a cantilever (with an indentation depth of 4 μm). From the force–distance curves obtained subsequently, the elastic modulus was determined (Figure 2B). The Young’s modulus obtained vertically on the apical surface of the pallium VZ was significantly greater (1412.3 ± 143.7 Pa, *n* = 11 pieces) than that obtained for the GE VZ (649.9 ± 111.2 Pa, *n* = 12 hemispheres) (*p* = 4.9 × 10^–5^, Mann–Whitney *U*-test), indicating that the apical surface is stiffer in the pallium than in the GE (Figure 2C).

### The Pallium–Ganglionic Eminence Difference in Apical Surface Stiffness Was Maintained Under Actomyosin-Inhibited Conditions

As a first approach to identifying the basis for the pallium-GE difference in the elastic modulus at the apical surface of the VZ, we coupled AFM indentation measurements with pharmacological experiments. We recently reported that actomyosin may partly contribute to the elastic modulus of the apical surface at the pallium (Nagasaka et al., 2016). We measured Young’s modulus under actomyosin-inhibited conditions using either blebbistatin or Y-27632 (a rho-kinase inhibitor). Under blebbistatin conditions, the elastic modulus in the pallium VZ was still greater (998.7 ± 138.8 Pa, *n* = 14 pieces) than that in the GE VZ (500.6 ± 34.5 Pa, *n* = 10 hemispheres) (*p* = 4.2 × 10^–5^, Mann–Whitney *U*-test) (Figure 2D). Under the Y-27632 condition, the elastic modulus in the pallium VZ was also greater (672.6 ± 54.3 Pa, *n* = 14 pieces) than that in the GE VZ (338.2 ± 31.1 Pa, *n* = 10 hemispheres) (Figure 2D). The decreases in Young’s moduli in response to both drugs were quite comparable between pallium and GE (50% decrease by Y-27632 and 30% decrease by blebbistatin) (Figure 2E). These results suggested that the stiffness at the apical surface of the VZ depends partly on actomyosin, but the observed pallium–GE difference may be explained mainly by other factors.

### The Stiffness of Dissociated Ventricular Zone Cells Was Comparable Between the Pallium and the Ganglionic Eminence

**FIGURE 3:**
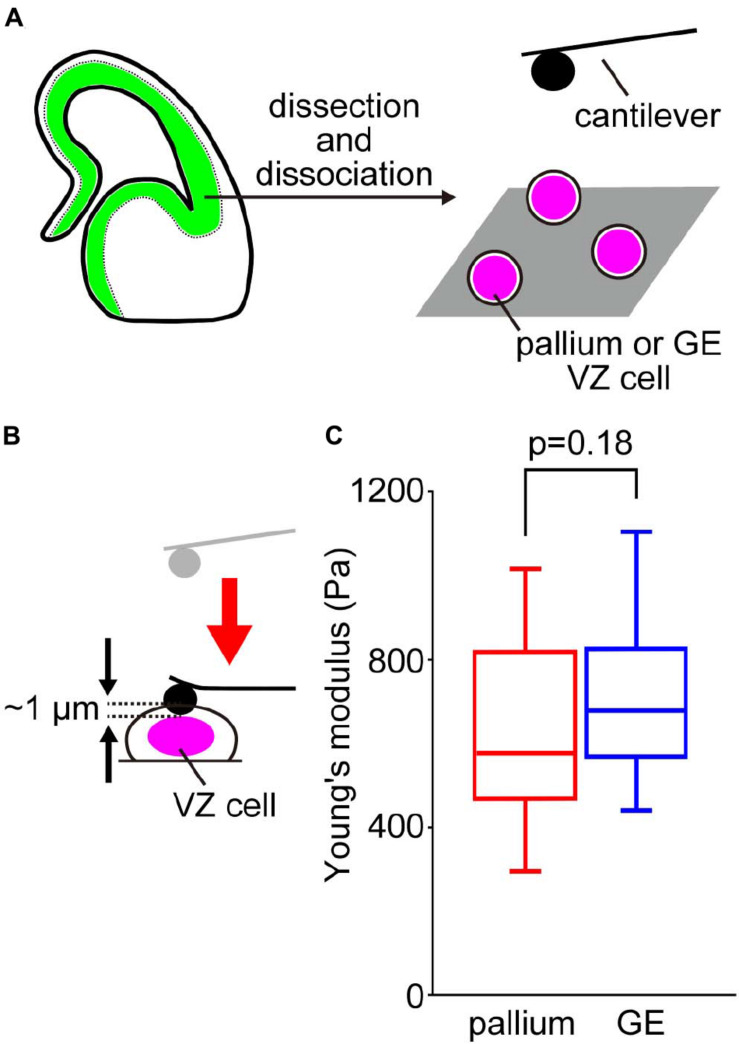
AFM measurements of cells dissociated from the VZ of the pallium and the GE. **(A)** Experimental design. **(B)** Illustration showing AFM indentation measurement of dissociated VZ cells. **(C)** Graph showing the Young’s moduli of pallial VZ cells (*n* = 50 cells) and GE VZ cells (*n* = 52 cells) (*p* = 0.18, Mann–Whitney *U*-test).

To determine whether the tissue-level difference in stiffness at the apical surface between pallium and GE can be explained by a possible difference in the stiffness of individual VZ cells of the two regions, we performed AFM indentation measurements on single, dissociated cells obtained from E13 mice (Figure 3A). Harvested cells were placed in culture dishes and pushed by spherical beads (5 μm in diameter) attached to the tip of a cantilever (with an indentation depth of 1 μm) (Figure 3B). We did not find significant differences between the Young’s modulus of pallium cells (690.9 ± 348.8 Pa, *n* = 50 cells) and that of GE cells (695.6 ± 159.5 Pa, *n* = 52 cells) (*p* = 0.18, Mann–Whitney *U*-test) (Figure 3C). This result suggests that the stiffness of single dissociated cells may not directly generate differential tissue-level stiffness at the apical surface.

### The Apical Surface Tension, as Revealed by Laser Ablation, Was Greater in the Ganglionic Eminence Ventricular Zone Than in the Pallial Ventricular Zone

**FIGURE 4:**
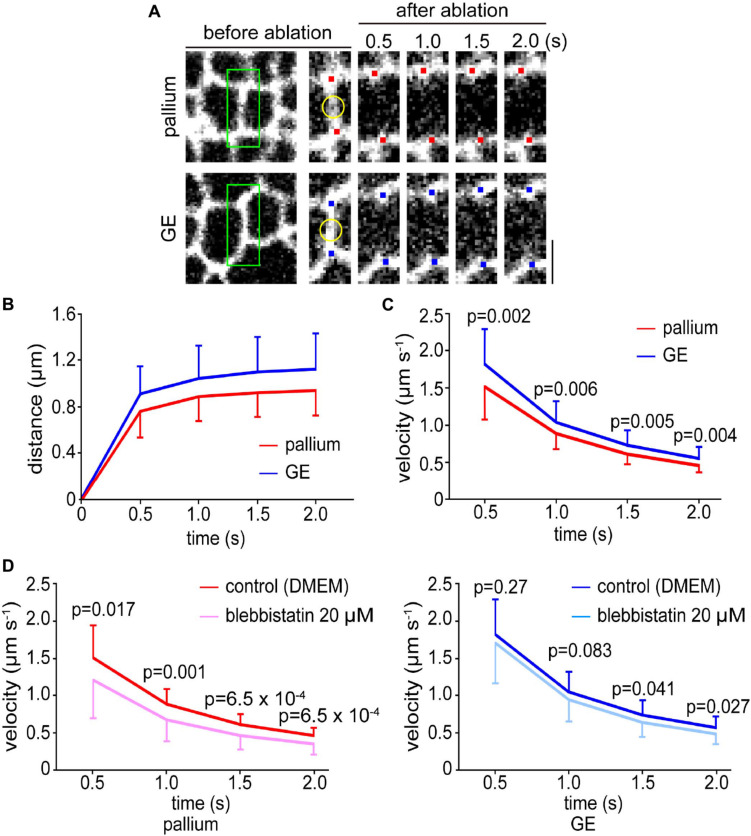
Laser ablation experiments were performed on the apical surfaces of the pallium and the GE. **(A)** *En face* live images of the apical surfaces of the pallial and GE walls prepared from an E13 ZO1-EGFP transgenic mouse. Mesh-like patterns are shown by apices of NPCs. A short-pulse laser was applied to the midpoint (marked in yellow) on the enlarged images of a boundary line (enclosed in green) formed by two polygonal apices of neighboring EGFP-labeled NPCs. Red (pallium) or blue (GE) dots highlight the vertices tracked. Scale bar, 2 μm. **(B)** Graph depicting the distance between the two tracked vertices at both ends of the laser-targeted side in pallium and GE. **(C)** Graph depicting the separation speed of two vertices at both ends of the laser-targeted side, showing that recoils were significantly quicker in GE (*n* = 52 sides) than in pallium (*n* = 35 sides) (*p* = 0.002 at 0.5 s; *p* = 0.006 at 1.0 s; *p* = 0.005 at 1.5 s; *p* = 0.004 at 2.0 s, Mann–Whitney *U*-test). **(D)** Graph showing the results of laser ablation in the presence of blebbistatin. In the pallium (left), the separation speed was significantly slower (*n* = 35 sides for control and *n* = 37 sides for blebbistatin) (*p* = 0.017 at 0.5 s; *p* = 0.001 at 1.0 s; *p* = 6.5 × 10^–4^, at 1.5 s; *p* = 6.5 × 10^–4^ at 2.0 s, Mann–Whitney *U*-test). In the GE (right), statistically significant differences were not observed at 0.5 s and 1.0 s (*n* = 52 sides for control and *n* = 37 sides for blebbistatin) and smaller than in the pallium at other time points (*p* = 0.27 at 0.5 s; *p* = 0.083 at 1.0 s; *p* = 0.041, at 1.5 s; *p* = 0.027 at 2.0 s, Mann–Whitney *U*-test).

Pharmacological experiments with blebbistatin and Y-27632 suggested that the dependence of the apical surface stiffness on the actomyosin system was comparable between the pallium and the GE. Apical actomyosin exhibits contractility to bend or curl the pallial wall (to increase its concavity), and laser ablation is useful for assessing such contractility of the pallial apical surface by measuring the recoils of cut edges (Okamoto et al., 2013; Nagasaka et al., 2016). Therefore, we compared the responses of the apical surface with laser ablation between the pallium and the GE. Using cerebral walls prepared from R26-ZO1-EGFP transgenic E13 mice in which the apices of VZ cells can be monitored live, we applied a short-pulse laser to the midpoint of a side (boundary line) formed by two polygonal apices of neighboring EGFP-labeled NPCs and measured the separation speed of two vertices at both ends of the laser-targeted side (*n* = 35 sides in pallium and *n* = 52 sides in GE) (Figure 4A and Supplementary Movie 1). Despite our initial expectation that similar recoiling patterns would be seen in pallium and GE (based on the commonly observed dependence on actomyosin in the AFM analysis), we found that recoils (separation of the tracked vertices) (Figure 4B) were significantly quicker (*p* = 0.002 at 0.5 s; *p* = 0.006 at 1.0 s; *p* = 0.005 at 1.5 s; *p* = 0.004 at 2.0 s, Mann–Whitney *U*-test) in the GE than in the pallium (Figure 4C).

### Blebbistatin Inhibited the Laser Ablation-Mediated Recoils Less Severely in the Ganglionic Eminence Than in the Pallium

We further sought to assess the degree of the dependence of the aforementioned recoiling of the laser-ablated apical surfaces on actomyosin. We, thus, performed comparative laser ablation in the presence of blebbistatin (20 μM). As shown in Figure 4D (left), recoiling of the pallial apical surface was significantly reduced by blebbistatin throughout the observation period (∼2.0 s) (*n* = 35 sides for control and *n* = 37 for blebbistatin) (*p* = 0.017 at 0.5 s; *p* = 0.001 at 1.0 s; *p* = 6.5 × 10^–4^ at 1.5 s; *p* = 6.5 × 10^–4^ at 2.0 s, Mann–Whitney *U*-test). However, the effect of blebbistatin to recoiling was overall smaller in the GE (*n* = 52 sides for control and *n* = 37 sides for blebbistatin) (*p* = 0.27 at 0.5 s; *p* = 0.083 at 1.0 s; *p* = 0.041 at 1.5 s; *p* = 0.027 at 2.0 s, Mann–Whitney *U*-test), most clearly at 0.5 and 1.0 s when statistically significant differences were not observed (Figure 4D, right), suggesting that the relative contribution of actomyosin to the laser ablation-mediated apical surface recoiling was different between the pallium and the GE. It was, therefore, speculated that the greater tangential tension on the apical surface of the GE than in the pallium (Figure 4A) might have been caused by a combination of an apical actomyosin-dependent mechanism like in the pallium and a possible GE-specific mechanism almost independent of apical actomyosin (discussed later). The observed enhanced recoiling responses in GE were reminiscent of the previously observed recoiling behaviors of the pallial apical surface that was experimentally overcrowded by aberrant cells via failed IKNM (Okamoto et al., 2013).

### Apical Surface Stiffness and Neural Progenitor Cell Apices Density Showed Correlations in Comparisons Between Different Developmental Stages in Both the Ganglionic Eminence and the Pallium

**FIGURE 5:**
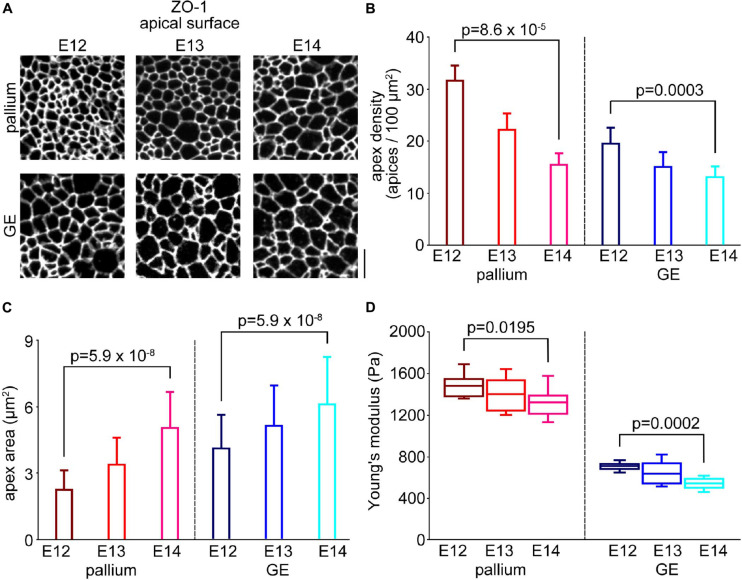
Comparison with the horizontal densities of endfeet between the pallium and the GE. **(A)** *En face* photomicrographs of the apical surface immunostained with anti-ZO1, showing junction meshwork patterns in the pallium and the GE from E12 to E14. Scale bar, 5 μm. **(B)** Graph showing the densities of apices in the pallium (*n* = 12 area from six embryos for E12, *n* = 12 area from seven embryos for E13, *n* = 12 area from seven embryos for E14) (*p* = 8.6 × 10^–5^, Steel–Dwass test) and the GE (*n* = 12 area from three embryos for E12, *n* = 12 area from three embryos for E13, *n* = 12 area from four embryos for E14) (*p* = 0.0003, Steel–Dwass test) from E12 to E14. **(C)** Graph showing the averaged area of the apex of each NPC endfoot in the pallium (*n* = 533 apices from eight embryos for E12, *n* = 511 apices from seven embryos for E13, *n* = 514 apices from seven embryos for E14) (*p* = 5.9 × 10^–8^, Steel–Dwass test) and GE (*n* = 524 apices from three embryos for E12, *n* = 530 apices from six embryos for E13, *n* = 514 apices from seven embryos for E14) (*p* = 5.9 × 10^–8^, Steel–Dwass test) cerebral walls from E12 to E14. **(D)** Graph showing the Young’s modulus measured vertically on the apical surface in the pallium (*n* = 10 pieces for E12, *n* = 11 pieces for E13, *n* = 15 pieces for E14) (*p* = 0.0195, Steel–Dwass test) and the GE (*n* = 12 pieces for E12, *n* = 12 pieces for E13, *n* = 10 pieces for E14) (*p* = 0.0002, Steel–Dwass test) from E12 to E14.

We previously showed in the developing mouse pallium that both the stiffness of the apical surface and the density of the NPC endfeet gradually decreased from E12 to E14 (Nagasaka et al., 2016). We could extend this analysis to comparisons between mice and ferrets, thereby finding that greater apical surface stiffness in ferrets than in mice was explained largely by the apical endfeet being denser in ferrets than in mice (Nagasaka et al., 2016). To determine whether the difference between the pallium and the GE can also be explained similarly, we carefully quantified the density of NPC apices (along with the area of each apex) in both the pallial and the GE walls using *en face* observation of walls immunostained with anti-ZO1 (Figure 5A) or those from R26-ZO1-EGFP transgenic mice.

In the GE, the density of the apices (per 100 μm^2^) gradually decreased between E12 and E14 (from 19.5 at E12 to 13.1 at E14, *p* = 0.0003, Steel–Dwass test) (Figure 5B). This was coupled with the enlargement of apices (4.1μm^2^ at E12 and 6.1 μm^2^ at E14, *p* = 5.9 × 10^–8^) (Figure 5C). Likewise, in the pallium, the density of the apices (per 100μm^2^) also gradually decreased between E12 and E14 (from 31.6 at E12 to 5.4 at E14, *p* = 8.6 × 10^–5^) (Figure 5B), and the apex area increased (from 2.3 μm^2^ at E12 to 5.1 μm^2^ at E14, *p* = 5.9 × 10^–8^) (Figure 5C). The Young’s modulus in the GE increased from E14 (541.0 ± 51.2 Pa, *n* = 10 hemispheres) to E12 (710.3 ± 37.0 Pa, *n* = 12 hemispheres) (*p* = 0.0002, Steel–Dwass test) (Figure 5D), and the Young’s modulus in the pallium also increased from E14 (1327.4 ± 129.9 Pa, *n* = 15 pieces) to E12 (1487.3 ± 99.9 Pa, *p* = 0.0195 *n* = 10 pieces). These results showed high correlations between the apices density and the apical surface stiffness in both the GE (*r* = 0.93) and the pallium (*r* = 0.99) during development from E12 to E14. Importantly, at any embryonic day during the E12–E14 period, the apex density and Young’s modulus together were greater in the pallium than in the GE (*p* = 6.1 × 10^–5^ for the density at E12, *p* = 4.0 × 10^–4^ for the density at E13, *p* = 0.02 for the density at E14, *p* = 1.7 × 10^–4^ for Young’s modulus at E12, *p* = 1.1 × 10^–4^ for Young’s modulus at E13, *p* = 7.2 × 10^–5^ for Young’s modulus at E14) (Supplementary Figure 1).

### Apical Surface F-Actin Meshwork Was Denser in the Pallium Than in the Ganglionic Eminence From Embryonic Days 12 to 14

**FIGURE 6:**
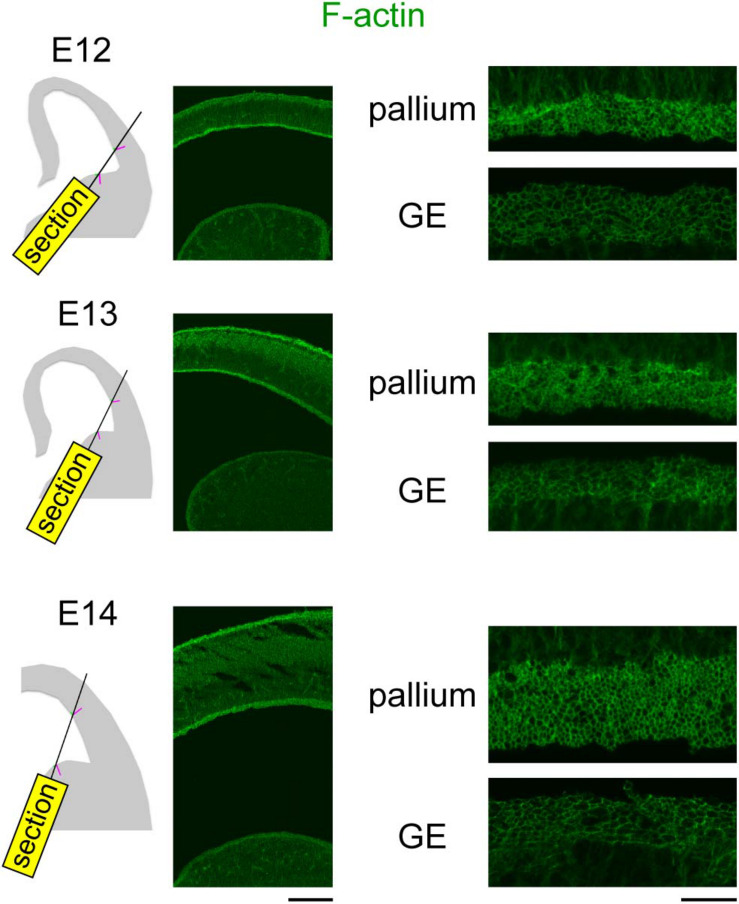
Photomicrographs showing confocal images of F-actin (green) obtained in the pallium and the GE at E12–E14. Plane of sectioning is schematically shown in the left panels: magenta line is perpendicular to the apical surface, while green lines represent F-actin^+^ apical surfaces simultaneously captured in the pallium and GE. Scale bar, 0.2 mm (left) and 20 μm (right).

To further gain a physical insight on the apical surface, we visualized F-actin, which is enriched at the apices of NPCs (Schenk et al., 2009; Shao et al., 2020). For simultaneous comparison between the pallium and the GE, sections oblique to the apical surface of both regions were prepared and stained with fluorescent labeled phalloidin (Figure 6). Low-power inspection revealed that a fluorescent-labeled “band” was much brighter at the concave-shaped pallial apical surface than along the convex-shaped GE apical surface. High-magnification observations showed that the thickness of each line in individual phalloidin^+^ F-actin rings (i.e., NPC apices) was greater in the pallium than in the GE, although it was not possible to determine whether this thickness difference (pallium > GE) can simply be explained by the difference in the apices of the NPCs (as shown in Figure 5), or other mechanisms such as a pallium-specific F-actin accumulation/enrichment might also be involved.

### Spontaneous Narrowing of the Apical Surface in Freshly Prepared Slices Was Milder in the Ganglionic Eminence Than in the Pallium

**FIGURE 7:**
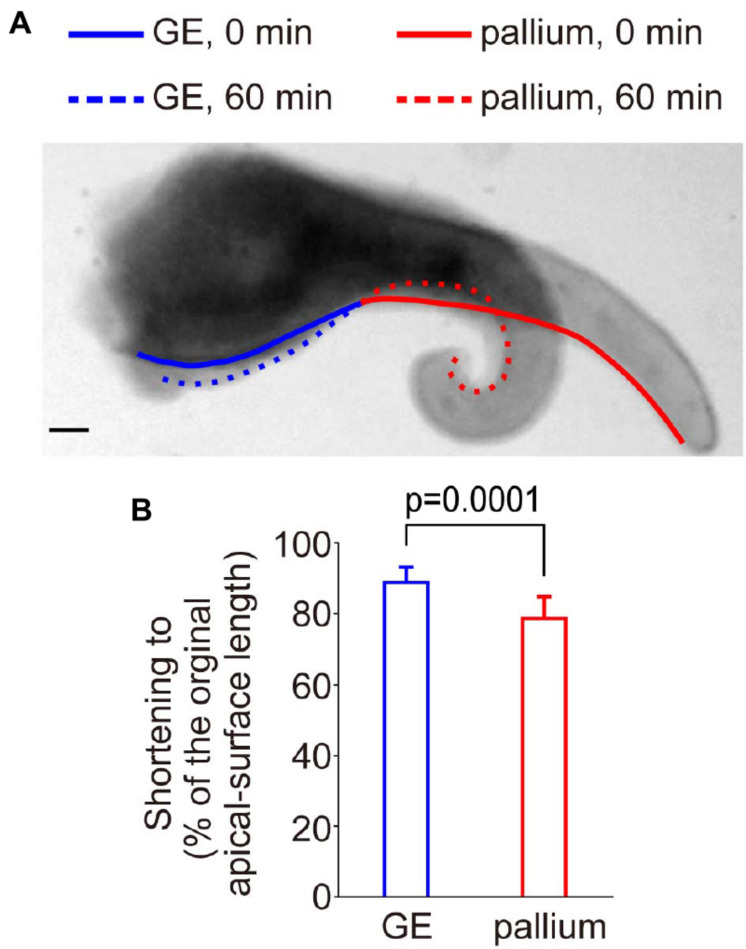
Spontaneous narrowing/shortening of the apical surface of sliced E12 cerebral walls. **(A)** An example of bending/curling of the pallium (i.e., shortening of the apical surface, red) accompanied with narrowing of the apical surface of GE (blue). Scale bar, 100 μm. **(B)** Graph comparing the shortening of the apical surface (from 0 to 60 min) between the pallium and the GE. The apical surface of the pallium (*n* = 6 slices) more significantly shortened than that of the GE (*n* = 6 slices) (*p* = 0.0001, Mann–Whitney *U*-test).

**FIGURE 8:**
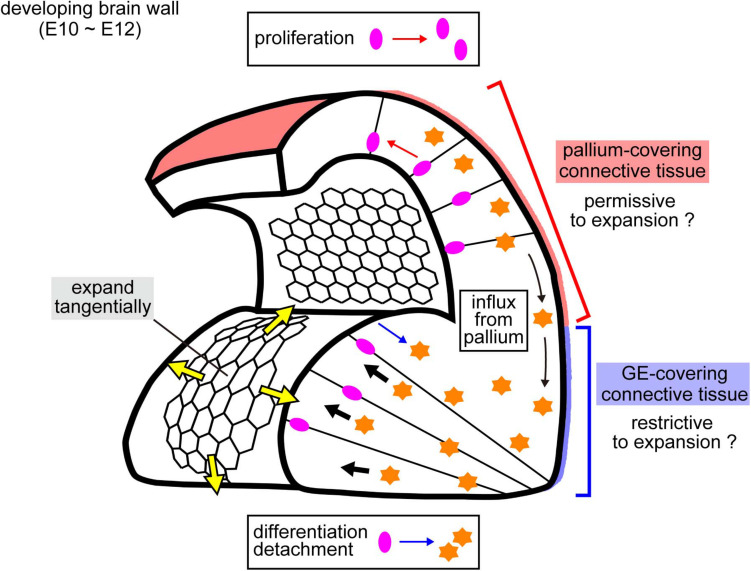
Schematic illustration of possible mechanical factors implicated in the emergence and growth of the two distinct cerebral wall morphologies. Laser ablation onto the apical surface revealed that the convex-shaped GE apical surface showed quicker recoils of edges than the pallial apical surface (Figure 4). This greater pretension on the GE apical surface (yellow arrows) could be caused by a pushing force (black arrows) arising from deeper parts. Cells detached from the apical surface (differentiating neurons and some NPCs), which seem to be more abundant in the GE than in the pallium (Smart, 1976; Smart and Smart, 1982; Pilz et al., 2013), and neurons immigrated from the pallium to the GE immigrant neurons (Saito et al., 2019), might act as an intramural stuffer. External factors would also be important, potentially playing a constricting role against lateral expansion of the cerebral wall, perhaps more strongly to the GE than to the pallium.

Not only laser ablation but also all kinds of surgical treatments including dissection/isolation and slicing of brains can allow tissues/cells to release residual stresses that had been stored *in vivo*, thereby inducing morphological changes measurable at mesoscopic scales. Bending/curling of freshly prepared cerebral pallial wall slices to the apical/concave direction is an exhibition of narrowing/shortening of the apical surface, which is observed within 60 min after slicing (Okamoto et al., 2013; Supplementary Movie 2). This apical surface narrowing is driven by actomyosin-dependent contractility (Nagasaka et al., 2016; Shinoda et al., 2018). In a previous study that forced the pallial apical surface to locally become convex through inducing subapical overcrowding (Okamoto et al., 2013), this apical bending/curling became less extensive than usual. Therefore, assessing the narrowing/shortening is another useful means to compare the convex (GE) and concave (pallial) cerebral wall apical surfaces. As summarized in Figure 7 (*n* = 6 cerebral wall slices consisting of both pallium and GE), shortening of the apical surface was significantly greater in the pallium than in GE (*p* = 0.0001, Mann–Whitney *U*-test), suggesting that the convex-shaped apical surface of the GE exhibits actomyosin-dependent narrowing less freely than the pallial apical surface does. This result, together with the aforementioned differential susceptibility of the apical surface recoiling to blebbistatin (i.e., recoiling of the GE apical surface was less dependent on actomyosin than that of the pallial apical surface) (Figures 4D,E), raises a possibility that the mechanical property of the GE apical surface might be affected passively by the neighboring cellular elements packed within GE (discussed below, see Figure 8).

## Discussion

### Factors Underlying the Differing Apical Surface Stiffness Between the Concave Pallium and the Convex Ganglionic Eminence

In this study, we compared the mechanical properties of the apical/inner surface of the developing (E12–E14) pallium and GE, two neighboring but distinct telencephalic regions that have formed (by E11) concave and convex inner-surface shapes, respectively (Figure 1). Through AFM indentation measurement, we first found that the Young’s modulus was higher at the concave-shaped apical surface of the pallium than at the convex-shaped apical surface of the GE (Figure 2). Further AFM analysis with pharmacological assessments (Figure 2) or on single dissociated NPCs (Figure 3) and laser ablation-mediated mechanical analysis (Figure 4) suggested that each dissociated NPC stiffness and the activity of actomyosin at the *in vivo* NPC apices were comparable between the pallium and the GE and that the apical surface tension was greater in the convex-shaped GE, which do not apparently account for the observed difference (pallium > GE) in the apical surface stiffness.

Through further examinations to determine how the pallial apical surface was stiffer than the GE apical surface, we finally found that the density of apices of NPCs in the VZ was greater in the apically stiffer pallium than in the GE (Figure 5) and found a similar correlation between the decreasing density of the apices between E12 and E14 and the declining apical surface stiffness in the same period in both the pallium and the GE (Figure 5). We previously showed that the apical endfeet of the NPCs were denser in the VZ of ferrets than in mice (Okamoto et al., 2014) and that stiffness measured at the apical surface of the VZ by AFM was much greater in ferrets than in mice (Nagasaka et al., 2016). These previous studies that focused on species differences and the present study that evaluated pallium–GE differences at embryonic stages when their apical concavity vs. convexity have already become evident together suggest that densification of apical NPC endfeet may be a universal contributor to an increase in the vertical stiffness at the apical surface of the VZ. However, whether differential F-actin meshwork (much denser in pallium than in GE, Figure 6) can simply be explained by the density of the NPC apices is unclear. It would be interesting to study whether pallial NPCs have unique mechanisms to condense F-actin to the apical surface for mechanical suitability associated with or required for concavity. Since the elastic property of the apical surface mechanically contributes to the initial migration of apically generated daughter cells in the pallium (Shinoda et al., 2018), it would also be interesting to investigate whether cell behaviors in the VZ of the GE, which are different in multiple aspects from those observed in the VZ of the pallium (Pilz et al., 2013), can be partly explained by the differing (pallium > GE) apical surface stiffness found in the present study.

### Possible Mechanisms Underlying the Formation/Emergence of an Apically Convex Ganglionic Eminence

Since the responsiveness (i.e., reduction) of the apical surface stiffness to pharmacological inhibition with blebbistatin or Y-27632 was similar between the pallium and the GE (Figure 2), the dependence of the apical surface stiffness on its actomyosin activity seems comparable between the pallium and the GE. Therefore, quicker recoiling upon laser ablation (and, thus, larger tangential pretension) in the apical surface of the GE than in that of the pallium (Figure 4) was unexpected and does not directly explain the observed pallium > GE apical surface stiffness difference. This greater apical recoiling upon ablation and pretension in the GE was reminiscent of the behavior shown by the apical surface of experimentally overcrowded pallial VZs (Okamoto et al., 2013). Autonomous narrowing of the apical surface was also different (pallium > GE) (Figure 7). These results obtained from multiple mechanical examinations may provide a hint for speculating possible mechanisms as to how the nascent subpallial (non-pallial) region that was initially (E10) apically concave then becomes an apically convex “eminence” by E11 (Figure 1).

If a cube-like box covered at its top by a flat rubber-like sheet is stuffed extensively with something (e.g., wadding), the sheet will bulge or balloon. By analogy, if the apical surface (reminiscent of an elastic sheet) is pushed by cells from the outer/basal side of the developing brain wall (i.e., from a deep layer away from the apical surface), such pushing would result in the convexity and tangential tension of the apical surface (Figure 8). However, if we similarly pack another box that is designed to automatically expand laterally, its rubber surface would not easily bulge due to lateral spreading of the wadding. The former and latter scenarios seem to apply better for normal GE and pallium, respectively, during the E10–E12 period (Figures 1, 8). Exceptionally, the pallial apical surface locally became convex at E12 when subapical overcrowding was induced by acute blocking of basal-ward IKNM (Okamoto et al., 2013). While the early embryonic brain wall and its apical surface laterally expand by the proliferation of undifferentiated NPCs, differentiating cells detach from the apical surface and move outward/basally, playing a stuffing role. Therefore, whether the mode of cell division in the VZ is purely proliferative (only giving rise to apically connected NPCs) or more differentiating (providing apically detaching and intramurally stuffing cells) may be a key intrinsic factor in shaping the developing brain wall (Figure 8). This idea is consistent with the production modes and cell movement observed in previous studies on GE NPCs and their daughter cells; differentiation and detachment from the apical surface occur earlier and more frequently in the GE than in the pallium (Smart, 1976; Smart and Smart, 1982; Pilz et al., 2013).

In addition, we recently showed that neurons born at the pallium migrate tangentially to the basal/outer part of the GE (Saito et al., 2019), which may also help thicken the GE (Figure 8). Since the extracellular matrix at the apical surface (aECM) can be a mechanical contributor to epithelial morphogenesis (Dong et al., 2014; Vuong-Brender et al., 2017), it would be interesting to examine whether (and if so, to what degree) the aECM also contributes to the apical surface stiffness and the pallium-GE difference. Furthermore, mechanical effects from the outer surrounding tissues (Shyer et al., 2013; Nelson, 2016) may also be important for brain morphogenesis because the abovementioned analogy of the GE to a non-expandable “box” depends on whether lateral expansion of the GE is restricted externally by surrounding connective tissues (Figure 8). Perhaps connective tissues dorsally covering the pallium might be more permissive to brain lateral expansion than ventral connective tissues over GE. The eminence-initiating (convexity-emerging) step (E10–E11) has not yet been reliably analyzed by our AFM system (due to difficulties in tissue preparations suitable for AFM analysis). Technical improvements will lead to better measurements of mechanical properties and thereby a deeper understanding of how mechanical forces are generated, sensed, and utilized by cells to form brain structures.

## Data Availability Statement

The raw data supporting the conclusion of this article will be made available by the authors, without undue reservation.

## Ethics Statement

The animal study was reviewed and approved by The Animal Care and Use Committee of Nagoya University.

## Author Contributions

AN performed all AFM measurements, pharmacological experiments, laser ablation experiments, and wrote the manuscript. TM designed the project. Both authors wrote the manuscript, contributed to the article, and approved the submitted version.

## Conflict of Interest

The authors declare that the research was conducted in the absence of any commercial or financial relationships that could be construed as a potential conflict of interest.

## Publisher’s Note

All claims expressed in this article are solely those of the authors and do not necessarily represent those of their affiliated organizations, or those of the publisher, the editors and the reviewers. Any product that may be evaluated in this article, or claim that may be made by its manufacturer, is not guaranteed or endorsed by the publisher.
