# Supplementary material for: Comparison of the Mechanical Properties Between the Convex and Concave Inner/Apical Surfaces of the Developing Cerebrum
Source: Front Cell Dev Biol. 2021 Jul 23;9:702068. doi: 10.3389/fcell.2021.702068 (PMC8343001; doi:10.3389/fcell.2021.702068)
Supplement: Supplementary file 1 [file Data_Sheet_1.docx]

Supplementary Material

**1) Supplemental figure**


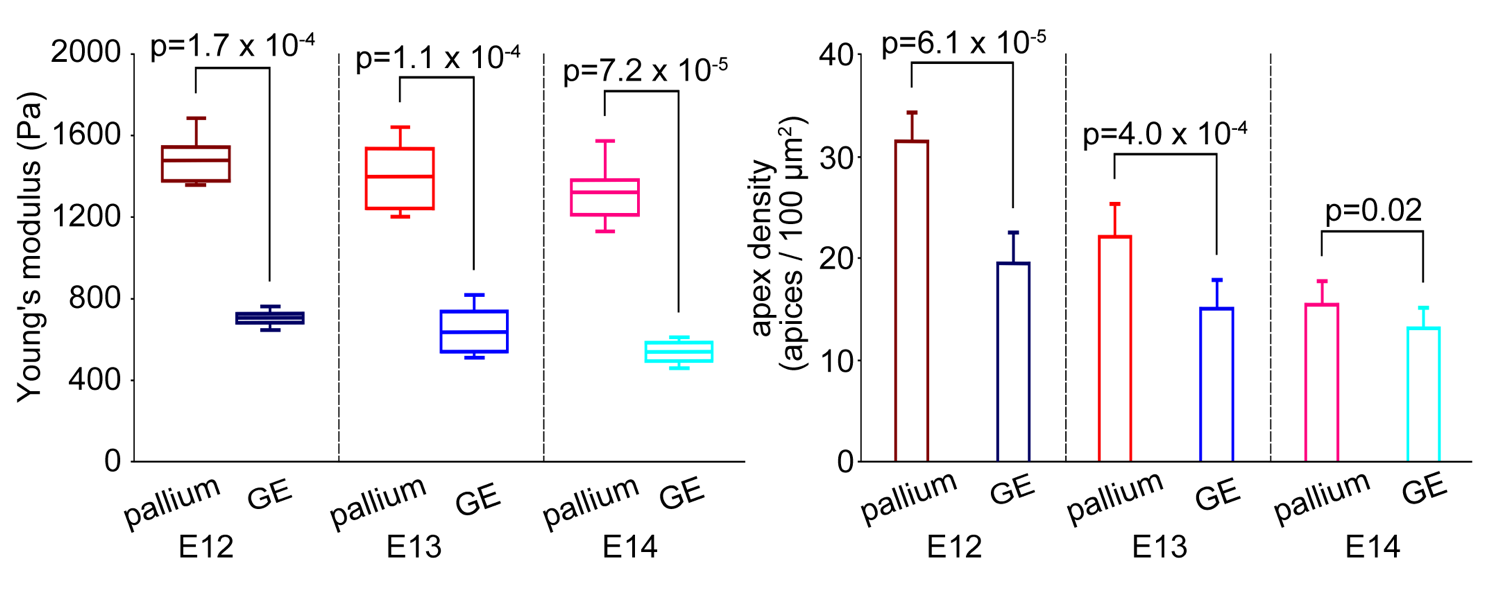
 **Figure S1.** Graph showing the Young’s modulus measured vertically on the apical surface and the densities of apices at each embryonic day during E12-E14 in the pallium and the GE (*p*= 1.7 x 10^-4^ for Young’s modulus at E12, *p*= 1.1 x 10^-4^ for Young’s modulus at E13, *p*= 7.2 x 10^-5^ for Young’s modulus at E14, *p*= 6.1 x 10^-5^ for density at E12, *p*= 4.0 x 10^-4^ for density at E13, *p*= 0.02 for density at E14, Mann-Whitney *U*-test).

**2) Supplemental movies**

**Supplemental Movie S1:** Laser ablation experiments were performed on the apical surfaces of the pallium (left) and the GE (right). Images were acquired 0.5 sec intervals. Movement of the vertices at both ends of the laser-targeted side was tracked, and results are summarized in Fig. 4.

**Supplemental Movie S2:** Spontaneous narrowing/shortening of the apical surface of sliced E12 cerebral walls. Images were acquired 30 sec intervals. Compare the shortening of the apical surface between the pallium and the GE, and results are summarized in Fig. 7.
